# Inhibition of cAMP/PKA Pathway Protects Optic Nerve Head Astrocytes against Oxidative Stress by Akt/Bax Phosphorylation-Mediated Mfn1/2 Oligomerization

**DOI:** 10.1155/2019/8060962

**Published:** 2019-11-06

**Authors:** Won-Kyu Ju, Myoung Sup Shim, Keun-Young Kim, Tae Lim Park, Sangphil Ahn, Genea Edwards, Robert N. Weinreb

**Affiliations:** ^1^Hamilton Glaucoma Center and Shiley Eye Institute, Viterbi Family Department of Ophthalmology, University of California San Diego, La Jolla, California, USA; ^2^Department of Ophthalmology, Duke University, Durham, North Carolina, USA; ^3^National Center for Microscopy and Imaging Research and Department of Neuroscience, University of California San Diego, La Jolla, California, USA

## Abstract

Glaucoma is characterized by a progressive optic nerve degeneration and retinal ganglion cell loss, but the underlying biological basis for the accompanying neurodegeneration is not known. Accumulating evidence indicates that structural and functional abnormalities of astrocytes within the optic nerve head (ONH) have a role in glaucomatous neurodegeneration. Here, we investigate the impact of activation of cyclic adenosine 3′,5′-monophosphate (cAMP)/protein kinase A (PKA) pathway on mitochondrial dynamics of ONH astrocytes exposed to oxidative stress. ONH astrocytes showed a significant loss of astrocytic processes in the glial lamina of glaucomatous DBA/2J mice, accompanied by basement membrane thickening and collagen deposition in blood vessels and axonal degeneration. Serial block-face scanning electron microscopy data analysis demonstrated that numbers of total and branched mitochondria were significantly increased in ONH astrocytes, while mitochondrial length and volume density were significantly decreased. We found that hydrogen peroxide- (H_2_O_2_-) induced oxidative stress compromised not only mitochondrial bioenergetics by reducing the basal and maximal respiration but also balance of mitochondrial dynamics by decreasing dynamin-related protein 1 (Drp1) protein expression in rat ONH astrocytes. In contrast, elevated cAMP by dibutyryl-cAMP (dbcAMP) or isobutylmethylxanthine treatment significantly increased Drp1 protein expression in ONH astrocytes. Elevated cAMP exacerbated the impairment of mitochondrial dynamics and reduction of cell viability to oxidative stress in ONH astrocytes by decreasing optic atrophy type 1 (OPA1), and mitofusin (Mfn)1/2 protein expression. Following combined treatment with H_2_O_2_ and dbcAMP, PKA inhibition restored mitochondrial dynamics by increasing mitochondrial length and decreasing mitochondrial number, and this promoted cell viability in ONH astrocytes. Also, PKA inhibition significantly promoted Akt/Bax phosphorylation and Mfn1/2 oligomerization in ONH astrocytes. These results suggest that modulation of the cAMP/PKA signaling pathway may have therapeutic potential by activating Akt/Bax phosphorylation and promoting Mfn1/2 oligomerization in glaucomatous ONH astrocytes.

## 1. Introduction

Glaucoma is a complex, multifactorial optic neuropathy characterized by a slow and progressive degeneration of the optic nerve, including retinal ganglion cell (RGC) axons, leading to visual impairment [1, 2]. The factors contributing to axon degeneration and astrocyte dysfunction in the optic nerve head (ONH) in primary open-angle glaucoma (POAG) are not well understood. Increasing evidence indicates that compromised astrocyte structure and function accompanied by RGC axon loss have been reported in the ONH of experimental glaucoma models and patients with POAG [3–8].

Astrocytes are the responsible cell type for many pathological alterations in the oxidative stress-mediated glaucomatous ONH degeneration [3, 6, 7, 9–13]. Since oxidative stress has long been thought to be an important pathophysiological mechanism in many neurodegenerative diseases including glaucoma [3, 10, 14–16], the activation of the cyclic adenosine 3′,5′-monophosphate (cAMP)/protein kinase A (PKA) signaling pathway exacerbates vulnerability to oxidative stress in ONH astrocytes [7]. The ubiquitous second messenger cAMP in the central nervous system contributes to numerous biological processes including cell growth and death [17–20]. Upon stimulation, cAMP synthesis and its degradation are tightly regulated by adenylyl cyclases and cyclic nucleotide phosphodiesterases (PDEs), respectively [18]. Accumulating evidence indicates that the increased level of cAMP is associated with the unstimulated glaucomatous ONH astrocytes from patients with POAG [21] as well as experimental glaucoma [7, 20]. Nevertheless, the precise cellular and molecular mechanism(s) of the activation of the cAMP/PKA signaling pathway underlying astrocyte dysfunction in glaucomatous ONH degeneration remains to be determined. In this regard, mitochondria have been suggested to have a key role.

In healthy cells, mitochondria are autonomous and morphologically dynamic organelles that structurally reflect a precise balance of ongoing dynamics, fission, and fusion, within a cell [22–24]. This balance is regulated by a family of dynamin-related GTPases that exert opposing effects. The mitofusins (Mfns) and optic atrophy type 1 (OPA1) are required for mitochondrial fusion, whereas dynamin-related protein 1 (Drp1) regulates mitochondrial fission [23–25]. Accumulating evidence indicates that compromised mitochondrial dynamics or function is associated with human ONH astrocytes from patients with POAG and rat ONH astrocytes exposed to oxidative stress *in vitro*, as well as glaucomatous ONH astrocytes in DBA/2J mice, an extensively characterized strain that spontaneously develops elevated intraocular pressure (IOP) [6, 9, 26–28]. Despite the widely appreciated glaucoma relevance of impaired mitochondrial dynamics and subsequent mitochondrial dysfunction [28–34], our understanding of the signaling mechanisms that impair mitochondrial structure and function in glaucomatous ONH astrocytes is still in its infancy. Accordingly, these mechanisms may be relevant to oxidative stress and/or cAMP/PKA pathway-mediated mitochondrial dysfunction [3, 10, 14–16].

In the present study, we investigate the impact of the activation of the cAMP/PKA pathway on mitochondrial dynamics in ONH astrocytes exposed to oxidative stress.

## 2. Materials and Methods

### 2.1. Animals

Pregnant Sprague-Dawley rats (250-300 g in weight; Envigo, Placentia, CA, USA) and adult female DBA/2J and DBA/2J-*Gpnmb^+^* (D2-*Gpnmb^+^*) mice (The Jackson Laboratory, Bar Harbor, ME, USA) were housed in covered cages, fed with a standard rodent diet ad libitum, and kept on a12 h light/12 h dark cycle. All procedures concerning animals were in accordance with the Association for Research in Vision and Ophthalmology Statement for the Use of Animals in Ophthalmic Vision Research and under protocols approved by Institutional Animal Care and Use Committee at the University of California, San Diego.

### 2.2. Reagents and Other Materials

Chemicals and other materials were obtained from the following sources: hydrogen peroxide (H_2_O_2_), dibutyryl-cAMP (dbcAMP), and H89 from Sigma, St. Louis, MO, USA; 8CPT-6phenyl-cAMP from Biolog; DMEM/F12 (1 : 1), MEM+GlutaMax™, 100x penicillin/streptomycin (Pen/Strep), and fetal bovine serum from Thermo Fisher Scientific, Waltham, MA, USA; Superscript™ II reverse transcriptase from Invitrogen; RNase inhibitor, dNTP mixture, and oligo (dT) from Promega; polymerase chain reaction (PCR) master mix, protein size marker, and SuperSignal® West Pico and Femto chemiluminescent substrates from Thermo Fisher Scientific; and Amersham Hybond™-P polyvinylidenedifluoride (PVDF) membrane and ECL™ Prime Western Blotting Detection Reagent from GE Healthcare (Chicago, IL, USA).

### 2.3. Tissue Preparation

Mice were anesthetized with intraperitoneal injection of a mixture of ketamine (100 mg/kg, Ketaset; Fort Dodge Animal Health, Fort Dodge, IA, USA) and xylazine (9 mg/kg, TranquiVed; Vedco, Inc., St. Joseph, MO, USA) before cervical dislocation. For electron microscopy (EM), the ONHs were dissected from the choroids and fixed in 2% paraformaldehyde and 2.5% glutaraldehyde (Ted Pella, Redding, CA, USA) in 0.15 M sodium cacodylate (pH 7.4, Sigma) at 37°C for 1 h.

### 2.4. Primary ONH Astrocyte Culture

Primary rat ONH astrocytes were prepared with minor modifications as previously described [7, 9]. Briefly, after euthanizing with CO_2_ gas, 10–20 pieces of ONH tissue were dissected from postnatal day 5 Sprague-Dawley rats and transferred to a 35 mm petri dish with 2 ml 0.2% bovine serum albumin/Dulbecco's PBS (DPBS). Under a dissecting microscope, remnant tissues were removed, and the ONH tissue was identified and dissected using microscissors and a sharp blade. The ONH tissues were minced and transferred to a 60 mm petri dish and conditioned with growth medium: MEM+GlutaMax™ supplemented with 10% FBS and 100x Pen/Strep and incubated at 5% CO_2_ at 37°C. After incubation for 1-2 weeks, the ONH explants were removed by 70 *μ*m cell strainers (BD Biosciences, San Diego, CA, USA). The cells that were grown from the ONH explants were plated in a 100 mm culture dish and cultured until 80% confluence. For further purification of the ONH astrocytes, the culture dish was shaken for 16 h at room temperature, followed by growth medium changing with serum free medium, and the cells were incubated for 24 h at 5% CO_2_ at 37°C. After removing nonadherent cells, the adherent ONH astrocytes were collected, centrifuged, and plated on poly-L-lysine-coated culture dishes.

### 2.5. Transmission Electron Microscopy (TEM)

For TEM analysis, ONH tissues were fixed via cardiac perfusion with solution at 37°C in 2% paraformaldehyde and 2.5% glutaraldehyde (Ted Pella) in 0.15 M sodium cacodylate (pH 7.4) and placed in precooled fixative on ice for 1 h as previously described [9, 29, 32]. The ONH tissues were embedded in Durcupan ACM resin (Fluka, St. Louis, MO, USA). Ultrathin (70 nm) sections were poststained with uranyl acetate and lead salts and evaluated with a JEOL 1200FX transmission EM operated at 80 kV (Tokyo, Japan). Images were recorded on film at 8000x magnification. For quantitative analysis, the number of astrocytic mitochondria was normalized to the total area occupied by astrocytes in each image, which was measured using ImageJ (National Institute of Health, Bethesda, MD, USA). Mitochondrial lengths were measured with ImageJ. The mitochondrial volume density, defined as the volume occupied by mitochondria divided by the volume occupied by the cytoplasm, was estimated using stereology as follows. A 112 × 112 square grid (112 × 112 chosen for ease of use with Photoshop) was overlaid on each image loaded in Photoshop (Adobe System, San Jose, CA, USA), and the mitochondria and cytoplasm lying under intercepts were counted. The relative volume occupied by mitochondria was expressed as the ratio of intercepts coinciding with this organelle relative to the intercepts coinciding with cytoplasm.

### 2.6. Serial Block-Face Scanning Electron Microscopy (SBEM)

For SBEM analysis, ONH tissues were washed with cacodylate buffer for 2 h at 4°C and then placed into cacodylate buffer containing 2 mM CaCl_2_ and 2% OsO_4_/1.5% potassium ferrocyanide as previously described [6, 32]. The tissues were left for 2 h at room temperature. After thorough washing in double distilled water, the tissues were placed into 0.05% thiocarbohydrazide for 30 min. The slices were again washed and then stained with 2% aqueous OsO_4_ for 1 h. The tissues were washed and then placed into 2% aqueous uranyl acetate overnight at 4°C. The tissues were washed with water at room temp and then stained with en bloc lead aspartate for 30 min at 60°C. The tissues were washed with water and then dehydrated on ice in 50%, 70%, 90%, 100%, and 100% ethanol solutions for 10 min at each step. The tissues were then washed twice in dry acetone and then placed into 50 : 50 Durcupan ACM : acetone overnight. The tissues were transferred to 100% Durcupan resin overnight. The tissues were then embedded and left in an oven at 60°C for 72 h. SBEM data was collected with a 3View unit (Gatan Inc., Pleasanton, CA, USA) installed on a Merlin field emission SEM (Carl Zeiss Microscopy, Thornwood, NY, USA). The ONH volumes were collected in 2.0 to 2.4 kV accelerating voltages, with a raster size of 24k × 24k and pixel dwell time of 0.5-1 *μ*s. The pixel sizes were 4.0-7.3 nm, depending on the raster size, and section thickness was 60-70 nm. Once a volume was collected, the histograms for the tissues throughout the volume stack were normalized to correct for drift in image intensity during acquisition. Digital micrograph files (.dm4) were normalized using Digital Micrograph and then converted to MRC format. The stacks were converted to eight bit, and volumes were manually traced for reconstruction and analysis using IMOD software (http://bio3d.colorado.edu/imod/) [35].

### 2.7. Cell Viability

Cell viability was measured using 3-[4, 5-dimethylthiazol-2yl]-2, 5-diphenyl tetrazolium bromide (MTT) according to the manufacturer's recommendations (Cell Proliferation Kit I; Roche Diagnostics, Basel, Switzerland) as previously described [7, 9, 32]. Briefly, ONH astrocytes were plated on a 96-well plate (0.5~1 × 10^4^ per well). After 48 h, the cells were treated with various reagents for 1 h and 10 *μ*l MTT stock solution was added to each well including the negative control. The cells were incubated for 4 h in a humidified atmosphere of a 5% CO_2_ incubator at 37°C, and 100 *μ*l of solubilization solution was added to dissolve the formazan crystals that remained in the wells. After overnight incubation at 5% CO_2_ at 37°C, the absorbance at 560 and 690 nm was measured with a microplate reader (SpectraMax; Molecular Devices, San Jose, CA, USA). Each set of data was collected from multiple replicate wells of each experimental group (*n* = 3).

### 2.8. Mitochondrial Morphology

Mitochondria in the ONH astrocytes were labeled by the addition of a red fluorescent mitochondrial dye (100 nM final concentration; MitoTracker Red CMXRos; Invitrogen, Carlsbad, CA, USA) to the cultures as previously described [6, 9] and were maintained for 20 min in a CO_2_ incubator. The cultures were subsequently fixed with 4% paraformaldehyde (Sigma) in DPBS for 30 min at 4°C. For three-dimensional (3D) reconstruction, images were obtained with an optical section separation (*z*-interval) of 0.25 *μ*m by an Olympus FluoView1000 (Olympus Corp., Tokyo, Japan). Isosurface rendition was obtained from the stack using Imaris 6.4.2 (Bitplane AG, Zurich, Switzerland).

### 2.9. Western Blot

Cells were harvested and lysed for 30 min on ice with a modified RIPA lysis buffer (150 mM NaCl, 1 mM EDTA, 1% NP-40, 0.1% SDS, 1 mM DTT, 0.5% sodium deoxycholate, and 50 mM Tris-Cl, pH 7.6), containing complete protease inhibitors. The lysates were centrifuged at 15000 g for 15 min, and the protein amounts in the supernatants were measured by Bradford methods. Proteins (5-10 *μ*g) were separated by SDS/PAGE and electrotransferred to the PVDF membrane. The membrane was blocked with 5% nonfat dry milk and PBS/0.1% Tween-20 (PBS-T) for 1 h, incubated with primary antibodies overnight at 4°C. Primary antibodies are rabbit polyclonal anti-Akt antibody (1 : 5000; Cell Signaling, Danvers, MA, USA), mouse monoclonal anti-phospho-Akt (Ser437) antibody (S473, 1 : 2000; Cell Signaling), mouse monoclonal anti-Bax antibody (N-20; 1 : 2000; Santa Cruz Biotechnology, Santa Cruz, CA, USA), rabbit polyclonal anti-phospho-Bax (Ser184) antibody (1 : 2000; Cell Signaling), mouse monoclonal anti-Drp1 antibody (1 : 5000; BD Biosciences), mouse monoclonal anti-OPA1 antibody (1 : 5000; BD Biosciences), mouse monoclonal anti-Mfn1 and anti-Mfn2 antibody (1 : 3000; Abcam, Cambridge, UK), and mouse monoclonal anti-actin antibody (1 : 100,000; Millipore, Burlington, MA, USA). After several washes in PBS-T, the membranes were incubated with peroxidase-conjugated goat anti-mouse or rabbit IgG (1 : 5000; Bio-Rad, Hercules, CA, USA) and developed using an enhanced chemiluminescence substrate system. The images were captured and quantified by using an ImageQuant™ LAS 4000 system (GE Healthcare Bio-Science), and the band densities were normalized to the band densities for actin.

### 2.10. Reverse Transcription- (RT-) PCR and Quantitative Real-Time RT-PCR

Total RNA was isolated from the cells using the RNeasy mini kit (Qiagen, Hilden, Germany), according to the manufacturer's protocol, as previously described [7]. cDNAs were synthesized from total RNAs with SuperScript II reverse transcriptase and oligo (dT) primers according to the manufacturer's protocols. RT-PCR was performed with cDNAs synthesized from 0.1 *μ*g of the total RNA of each cell as a template and specific primers (Table 1). RT-PCR products were electrophoresed on a 2% agarose gel and visualized by ethidium bromide staining. For the quantification of the relative mRNA expressions of each group, real-time PCR was carried out using a MX3000P real-time PCR system (Stratagene, San Diego, CA, USA) as follows. cDNAs were amplified using iQ™ SYBR Green super-mix (Bio-Rad) and the specific primers for 40 cycles (initial incubation at 50°C for 2 min and then at 95°C for 10 min, and 40 cycles (95°C for 15 sec, 55°C for 1 min, and 72°C for 1 min)). Output data were obtained as Ct values and the differential mRNA expression of each gene among samples was calculated using the comparative Ct method. GAPDH mRNA, an internal control, was amplified along with the target genes, and the Ct value of GAPDH was used to normalize the expression of target genes.

### 2.11. Mitochondrial Respiration

Cells (2 × 10^4^ per well) were seeded into Seahorse XF24-well plates approximately 24 h before the measurement. Oxygen consumption rate (OCR) was measured using an XF24 analyzer (Agilent, Santa Clara, CA, USA) as previously described [36]. After measuring the basal respiration, oligomycin (2 *μ*g/ml, Sigma), an inhibitor of adenosine triphosphate (ATP) synthesis; carbonyl cyanide 4-(trifluoromethoxy) phenylhydrazone (FCCP; 0.3 *μ*M, Sigma), the uncoupler; and rotenone (2 *μ*M, Sigma), an inhibitor of mitochondrial complex I, were sequentially added to measure maximal respiration.

### 2.12. Statistical Analyses

Data were presented as the mean ± S.D. Comparison of experimental conditions was evaluated using the two-tailed unpaired Student's *t*-test between groups or one-way analysis of variance followed by Tukey's post hoc test among groups. *P* < 0.05 was considered to be statistically significant.

## 3. Results

### 3.1. Mitochondrial Fission and Loss in Glaucomatous ONH Astrocytes

**Figure 1:**
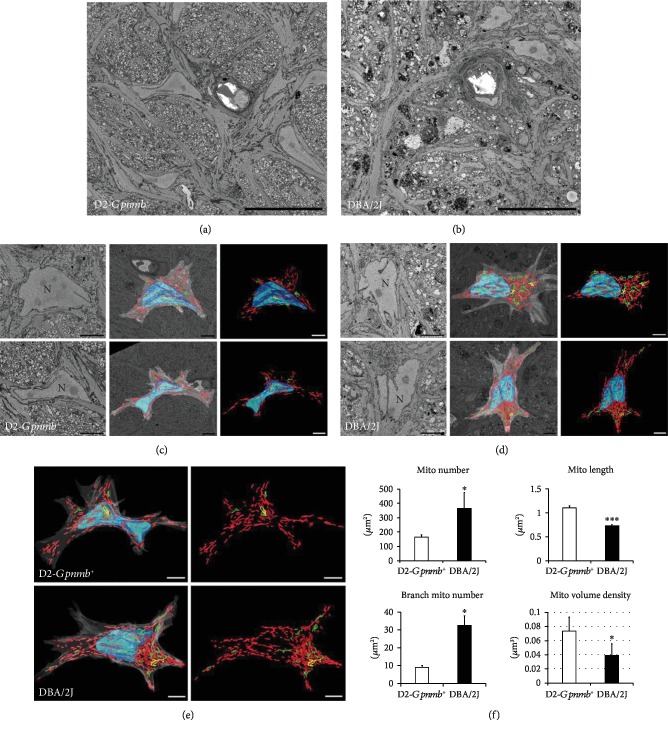
Mitochondrial fission and loss in the ONH astrocytes of the glial lamina in glaucomatous DBA/2J mice. (a) A representative SBEM micrograph showing normal healthy morphology of astrocytes, axons, and a blood vessel in the glial lamina of aged matched nonglaucomatous control D2-*Gpnmb^+^* mice. (b) A representative SBEM micrograph showing a significant loss of astrocytic processes and axonal degeneration as well as basement membrane thickening and collagen deposition in the blood vessels in the glial lamina of glaucomatous DBA/2J mice. (c–e) Representative 3D reconstruction micrographs showed separated (red) and branched (green) mitochondria, as well as the nucleus (blue) in ONH astrocyte somas in both groups. Scale bars, 10 *μ*m. (f) Quantitative analysis of mitochondrial number, lengths, and volume density as well as branched mitochondrial number in ONH astrocyte somas. Data are shown as the mean ± SEM (SBEM, *n* = 2 mice/group; *n* = 4 ONH astrocytes/group). ∗ and ∗∗∗ denote *P* < 0.05 and *P* < 0.001, respectively.

**Figure 2:**
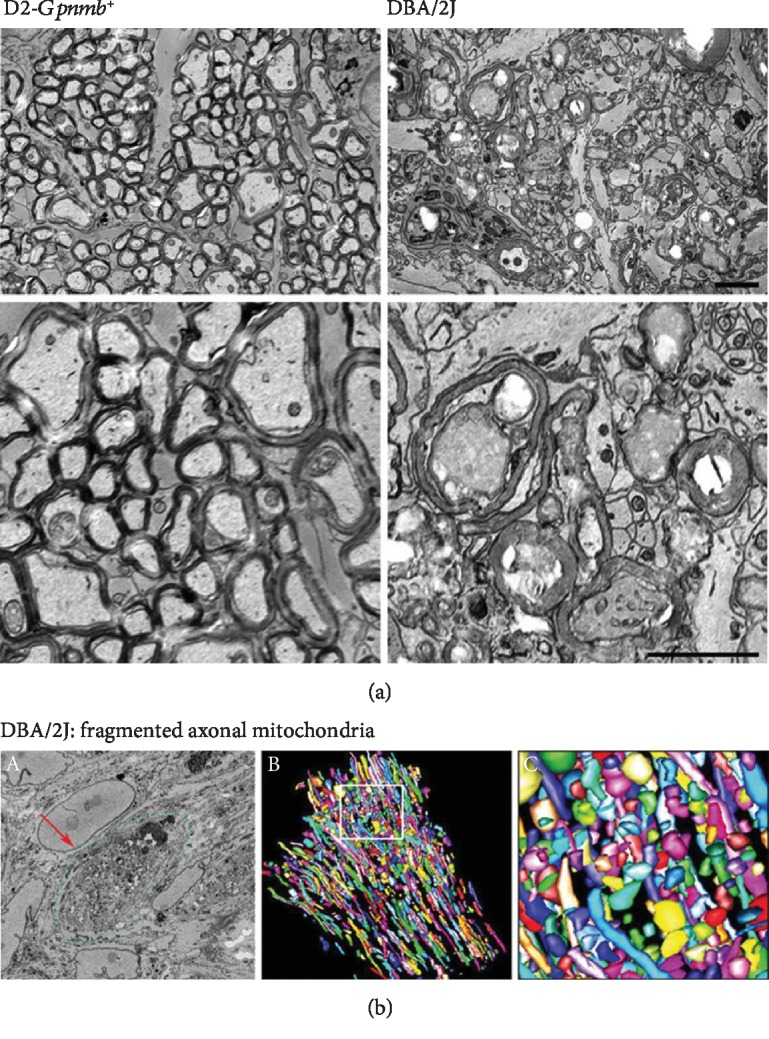
Mitochondrial fission in axonal degeneration of the glial lamina in glaucomatous DBA/2J mice. (a) Representative TEM micrographs showed normal healthy morphology of axons and myelins, as well as astrocytic processes in the glial lamina of aged matched nonglaucomatous control D2-*Gpnmb^+^* mice. However, representative TEM micrographs showed a significant loss of axon and myelins as well as disrupted astrocytic processes in the glial lamina of glaucomatous DBA/2J mice. Scale bars, 20 nm. (b) Representative 3D reconstruction micrographs from a SBEM data set showed highly fragmented mitochondria (several colors) in the axons of the glial lamina in glaucomatous DBA/2J mice. The arrow indicates damaged axon bundle. Scale bars, 10 *μ*m (C) and 1 *μ*m (A and B).

Structural and functional abnormalities have been implicated in ONH astrocytes with glaucoma progression [6, 9, 26, 28, 37]. The SBEM data set showed degenerative structural changes in the astrocytes of the glial lamina in glaucomatous DBA/2J mice (Figure 1). Compared with nonglaucomatous control D2-*Gpnmb^+^* mice (Figure 1(a)), glaucomatous ONH astrocytes showed a significant loss of astrocytic processes in the glial lamina, accompanied by basement membrane thickening and collagen deposition in the blood vessel, as well as axonal degeneration (Figure 1(b)). Using 3D reconstruction segmentations from the SBEM stack, our results revealed separated and branched mitochondria, as well as nucleus in astrocyte somas in both groups (Figures 1(c)–1(e)). Quantitative analysis of mitochondrial morphology showed that numbers of total mitochondria and branched mitochondria were significantly increased in glaucomatous ONH astrocytes compared with D2-*Gpnmb^+^* mice (Figure 1(f)). However, mitochondrial lengths and volume density were significantly decreased in glaucomatous ONH astrocytes compared with D2-*Gpnmb^+^* mice (Figure 1(f)). In good agreement with these results, compared with nonglaucomatous D2-*Gpnmb^+^* mice, TEM or SBEM data set analyses from glaucomatous DBA/2J mice consistently showed degenerative axons and myelins in the ON (Figure 2(a)) and mitochondrial fragmentation in the ONH axons (Figure 2(b)) [32].

### 3.2. Oxidative Stress Impairs Mitochondrial Bioenergetics and Alters Mitochondrial Dynamics by Decreasing Drp1 Protein Expression in ONH Astrocytes

**Figure 3:**
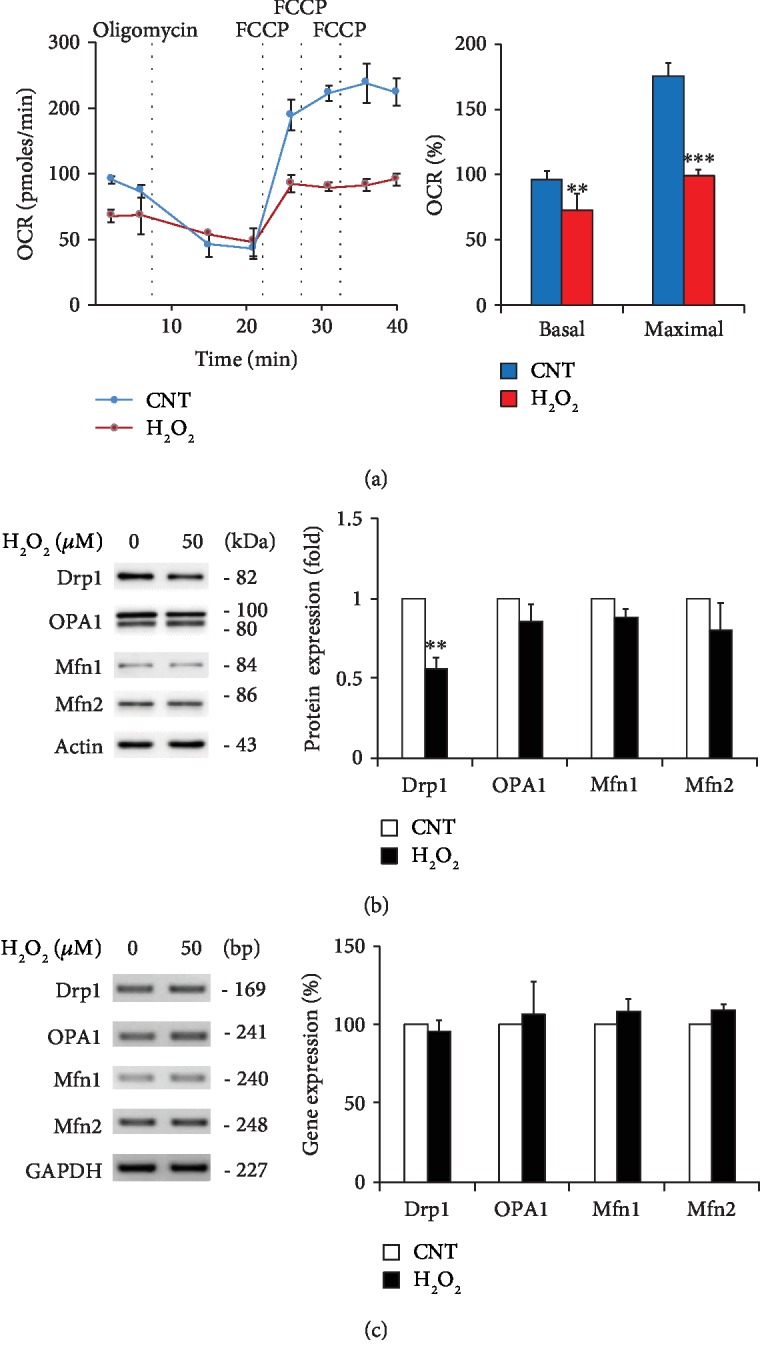
Effects of oxidative stress on mitochondrial bioenergetics and dynamics in ONH astrocytes. (a) OCR changes in ONH astrocytes treated with H_2_O_2_ (50 *μ*M) for 1 h. Oligomycin A and FCCP were sequentially added at the indicated time point. Basal respiration indicates the starting basal OCR and the value which was set to 100%. Maximum respiration represents the ratio between FCCP uncoupled OCR and basal OCR. (b) Western blot analyses for the protein expression of Drp1, OPA1, and Mfn1 and Mfn2 in ONH astrocytes treated with H_2_O_2_ (50 *μ*M) for 1 h. Note that oxidative stress induced loss of Drp1 protein expression in ONH astrocytes. (c) Real-time RT-PCR analysis for the mRNA expression of *Drp1*, *OPA1*, and *Mfn1* and *Mfn2* genes in ONH astrocytes treated with H_2_O_2_ (50 *μ*M) for 1 h. Note that oxidative stress did not change gene expression. For each determination, the expression levels of mRNAs and proteins in controls were normalized to a value of 100% and 1.0, respectively. Data are shown as the mean ± S.D. (*n* = 3). ∗∗ and ∗∗∗ denote *P* < 0.01 and *P* < 0.001, respectively.

Oxidative stress triggers mitochondrial dysfunction in ONH astrocytes by altering the oxidative phosphorylation (OXPHOS) complex (Cx), reducing cellular ATP production, and increasing reactive oxygen species (ROS) generation [9]. Using the Seahorse XF24 Flux Analyzer, we assessed mitochondrial respiration by directly measuring the OCR in ONH astrocytes following oxidative stress induced by H_2_O_2_ (50 *μ*M) as previously reported [7, 9]. Bioenergetic profile analysis showed that oxidative stress significantly decreased the basal and maximal respirations by 72 ± 10 and 99 ± 5 pmols/min compared with control cells by 96 ± 6 and 175 ± 13 pmols/min, respectively (Figure 3(a)). Of interest, our results demonstrated that oxidative stress significantly decreased total Drp1 protein expression, whereas there were no significant changes of the expression levels of OPA1 as well as Mfn1 and Mfn2 proteins in ONH astrocytes (Figure 3(b)). In addition, quantitative real-time RT-PCR analysis demonstrated that there were no statistically significant differences in the expression levels of *Drp1* and *OPA1* as well as *Mfn1* and *Mfn2* genes in ONH astrocytes exposed to H_2_O_2_ compared with control cells (Figure 3(c)).

### 3.3. Elevated Intracellular cAMP Alters Mitochondrial Dynamics by Increasing Drp1 Protein Expression in ONH Astrocytes

**Figure 4:**
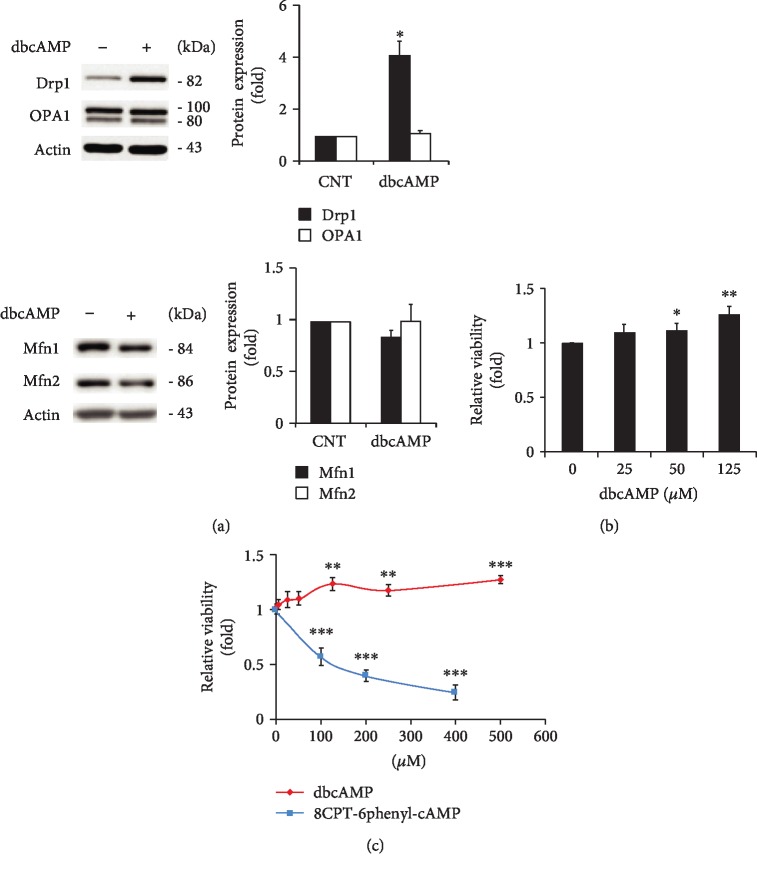
Effects of elevated intracellular cAMP on mitochondrial bioenergetics and dynamics in ONH astrocytes. (a) Western blot analyses for the protein expression of Drp1, OPA1, and Mfn1 and Mfn2 in ONH astrocytes treated with dbcAMP (100 *μ*M) for 1 h. Note that elevated intracellular cAMP induced increase of Drp1 protein expression in ONH astrocytes. (b, c) Cell viability/mitochondrial activity analysis using MTT assay in ONH astrocytes treated with dbcAMP or 8CPT-6phenyl-cAMP for 1 h. Note that cell viability was significantly decreased in ONH astrocytes by 8CPT-6phenyl-cAMP treatment in a dose-dependent manner, while dbcAMP treatment slightly increased cell viability. Data are shown as the mean ± S.D. (*n* = 3). ∗, ∗∗, and ∗∗∗ denote *P* < 0.05, *P* < 0.01, and *P* < 0.001 (two-tailed unpaired Student's *t-*test), respectively. For each determination, the cAMP level and protein expression in controls were normalized to the protein contents and a value of 1.0, respectively. Data are shown as the mean ± S.D. (*n* = 3). ∗, ∗∗, and ∗∗∗ denote *P* < 0.05, *P* < 0.01, and *P* < 0.001, respectively.

Our previous study demonstrated that forskolin-induced intracellular cAMP elevation impaired ONH astrocytes and prolonged elevation of cAMP-induced caspase-3-mediated cell death in ONH astrocytes [7]. To determine whether elevated cAMP alters mitochondrial dynamics and cell viability/mitochondrial activity in ONH astrocytes, we treated cells with dbcAMP, which is a cell-permeable analog of cAMP that mimics the action of endogenous cAMP in cultured ONH astrocytes. We observed that dbcAMP treatment significantly increased total Drp1 protein expression in ONH astrocytes, whereas the expression levels of OPA1 as well as Mfn1 and Mfn2 protein were not significantly changed in ONH astrocytes (Figure 4(a)). We further observed that dbcAMP treatment increased cell viability/mitochondrial activity dose-dependently in ONH astrocytes by MTT assay (Figure 4(b)). To confirm whether exogenous cAMP treatment itself reduces cell viability/mitochondrial activity or not, we further treated with 8CPT-6phenyl-cAMP, a natural cAMP analog with enhanced cell permeability and potent activator of PKA, and compared it to dbcAMP treatment in ONH astrocytes. We found that cell viability/mitochondrial activity was significantly decreased in ONH astrocytes by 8CPT-6phenyl-cAMP treatment in a dose-dependent manner, while dbcAMP treatment slightly increased cell viability (Figure 4(c)).

### 3.4. Elevated Intracellular cAMP Exacerbates Abnormality of Mitochondrial Dynamics in ONH Astrocytes Exposed to Oxidative Stress

**Figure 5:**
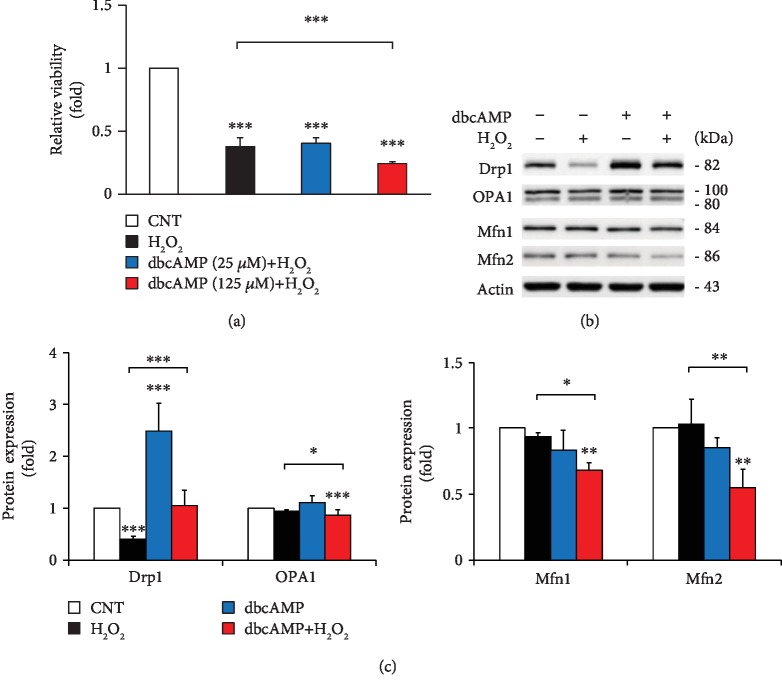
Effects of elevated intracellular cAMP on mitochondrial dynamics in ONH astrocytes exposed to oxidative stress. ONH astrocytes exposed to H_2_O_2_ (50 *μ*M) were cotreated with dbcAMP (25 or 125 *μ*M) for 1 h. (a) Cell viability/mitochondrial activity analysis using MTT assay in ONH astrocytes. Note that elevated intracellular cAMP accelerated loss of cell viability in ONH astrocytes exposed to H_2_O_2_ compared with ONH astrocytes exposed to H_2_O_2_ alone. (b, c) Western blot analyses for the protein expression of Drp1, OPA1, and Mfn1 and Mfn2. Note that elevated intracellular cAMP induced loss of total OPA1 and Mfn1 and Mfn2 protein expression in ONH astrocytes exposed to H_2_O_2_ compared with ONH astrocytes exposed to H_2_O_2_ alone. For each determination, the cell viability and protein expression in controls were normalized to a value of 1.0. Data are shown as the mean ± S.D. (*n* = 3). ∗, ∗∗, and ∗∗∗ denote *P* < 0.05, *P* < 0.01, and *P* < 0.001, respectively.

Because elevated intracellular cAMP exacerbated vulnerability to oxidative stress in ONH astrocytes *in vitro* and was also associated with compromised ONH astrocytes in glaucomatous DBA/2J mice *in vivo* [7], we further investigated whether elevated cAMP exacerbates abnormality of mitochondrial dynamics to oxidative stress in ONH astrocytes. We observed that lower concentration (25 *μ*M) of dbcAMP together with H_2_O_2_ (50 *μ*M) did not change the cell viability/mitochondrial activity in ONH astrocytes compared with H_2_O_2_ treatment alone. However, higher concentration (125 *μ*M) of dbcAMP with H_2_O_2_ (50 *μ*M) significantly decreased the cell viability/mitochondrial activity compared with H_2_O_2_ treatment alone (Figure 5(a)). Although dbcAMP is a membrane permeable cAMP analog, the intracellular concentration of dbcAMP reaches only 3 to 5% of dbcAMP applied extracellularly in the cell culture media, and importantly, only a small amount (~ 0.7 *μ*M) of N^6^-monobuturyl-cAMP, a biologically active derivative of dbcAMP, could be detected when dbcAMP was applied in extracellular concentrations of 1 mM [38, 39]. In addition, the order of antiproliferative activity correlated with the membrane permeability determined in the HPLC experiments (8‐CPT‐cAMP > dbcAMP > 8‐Br‐cAMP) [38]. These data could explain why the lower amount of dbcAMP (25 *μ*M) does not reduce cell viability after oxidative stress in contrast to the higher amount of dbcAMP (100 *μ*M). We have used the concentration (100 *μ*M) of dbcAMP to perform a combined treatment with H_2_O_2_ in the present study (Figures 5(b) and 5(c)). Intriguingly, we observed that elevated cAMP induced by dbcAMP restored total Drp1 protein expression in ONH astrocytes exposed to H_2_O_2_ up to the normal level like nontreated control cells compared with control cells exposed to H_2_O_2_ alone (Figures 5(b) and 5(c)), whereas elevated cAMP significantly decreased OPA1 as well as Mfn1 and Mfn2 protein expression in ONH astrocytes exposed to H_2_O_2_ (Figures 5(b) and 5(c)).

### 3.5. PKA Inhibition Restores Mitochondrial Dynamics in ONH Astrocytes against Oxidative Stress and cAMP Elevation

**Figure 6:**
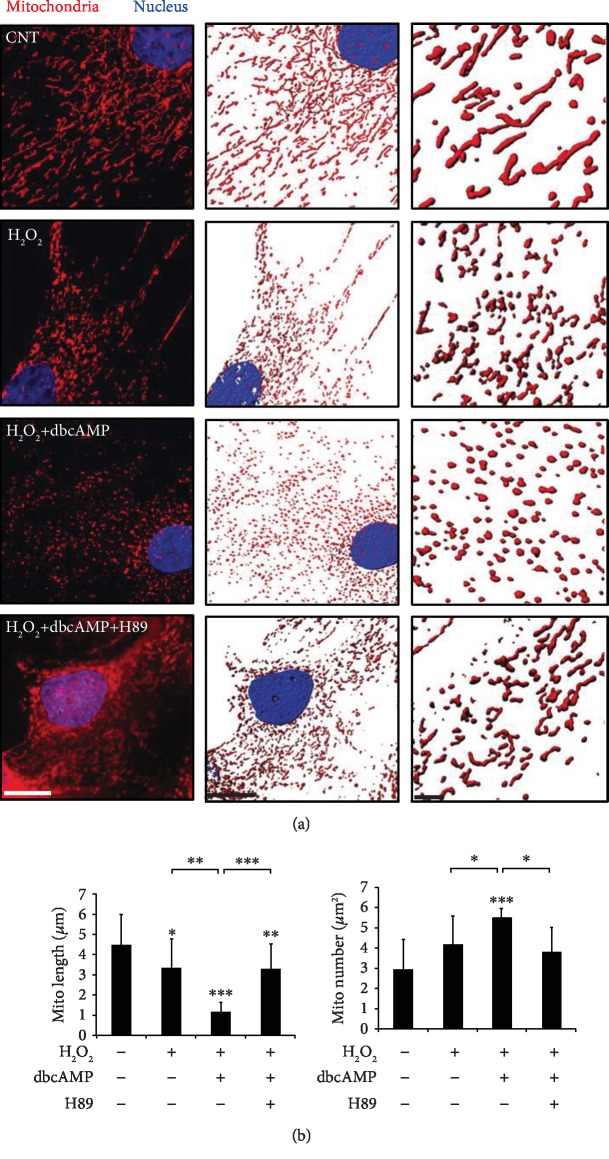
Effects of PKA inhibition on mitochondrial morphology in ONH astrocytes against combined oxidative stress and cAMP elevation. ONH astrocytes exposed to H_2_O_2_ (50 *μ*M) were cotreated with dbcAMP (100 *μ*M) and/or H89 (10 *μ*M) for 1 h. (a) MitoTracker Red staining of mitochondria in ONH astrocytes treated with H_2_O_2_ and/or dbcAMP. Scale bars, 10 *μ*m. (b) Quantitative analysis of mitochondrial number and lengths in ONH astrocyte somas. Note that PKA inhibition restores mitochondrial number and lengths in ONH astrocytes against combined oxidative stress and cAMP elevation. Data are shown as the mean ± S.D. (*n* = 608, 858, 937, and 516 mitochondria for control, H_2_O_2_, H_2_O_2_+cAMP, and H_2_O_2_+cAMP+H89, respectively). ∗∗ and ∗∗∗ denote *P* < 0.01 and *P* < 0.001, respectively.

We next determined the effect of PKA inhibition on mitochondrial dynamics in ONH astrocytes against oxidative stress combined with cAMP elevation. Using H89, a PKA inhibitor, we inhibited the PKA pathway in ONH astrocytes exposed to H_2_O_2_ and dbcAMP for 1 h. We further determined mitochondrial morphology in ONH astrocytes using MitoTracker Red staining, a marker for mitochondria [6, 9] and quantified mitochondrial length and number. As shown in Figure 5, our results demonstrated that control ONH astrocytes contained classic elongated tubular mitochondria. However, we observed the induction of mitochondrial fission in ONH astrocytes exposed to H_2_O_2_ compared with control cells (Figure 6(a)). We observed that intracellular cAMP elevation promoted an increase in mitochondrial fission and contained small rounded mitochondria in ONH astrocytes exposed to H_2_O_2_ compared with ONH astrocytes exposed to H_2_O_2_ alone (Figure 6(a)). Notably, this extensive mitochondrial fission by oxidative stress combined with cAMP elevation restored the mitochondrial morphology of ONH astrocytes by PKA inhibition like ONH astrocytes exposed to H_2_O_2_ alone (Figure 6(a)). Consistent with our data of worsening ONH astrocyte viability, quantitative analyses showed that oxidative stress combined with cAMP elevation induced a more severe decrease of mitochondrial length, but an increase of mitochondrial number in ONH astrocytes exposed to H_2_O_2_ compared with control cells exposed to H_2_O_2_ alone (Figure 6(b)). Of note, PKA inhibition significantly restored the mitochondrial length and number in ONH astrocytes against oxidative stress combined with cAMP elevation up to the level like ONH astrocytes exposed to H_2_O_2_ alone (Figure 6(b)).

### 3.6. PKA Inhibition Promotes Oligomerization of Mfn1 and Mfn2 and Phosphorylation of Akt and Bax in ONH Astrocytes against Oxidative Stress and cAMP Elevation

**Figure 7:**
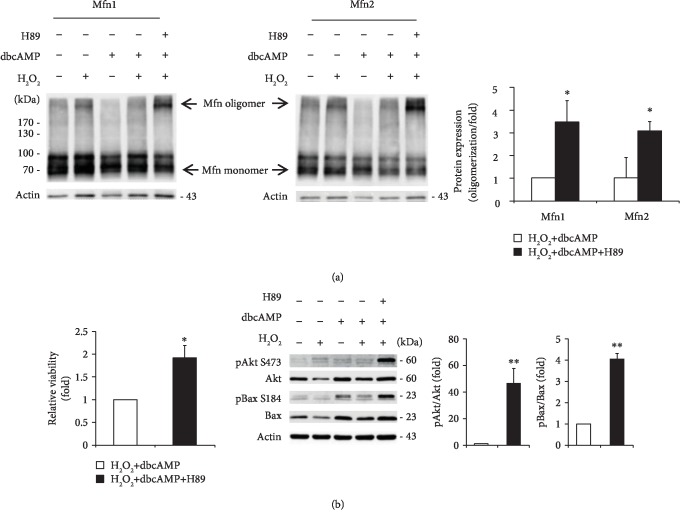
Effects of PKA inhibition on Mfn1 and Mfn2 oligomerization, and Akt and Bax phosphorylation in ONH astrocytes against combined oxidative stress and cAMP elevation. ONH astrocytes exposed to H_2_O_2_ (50 *μ*M) were cotreated with dbcAMP (100 *μ*M) and/or H89 (10 *μ*M) for 1 h. (a) Western blot analyses for the oligomerization of Mfn1 and Mfn2 proteins in ONH astrocytes. Note that PKA inhibition induced a significant increase of Mfn1 and Mfn2 protein oligomerization in ONH astrocytes treated with combined H_2_O_2_ and dbcAMP compared with ONH astrocytes treated with combined H_2_O_2_ and dbcAMP. (b) Cell viability/mitochondrial activity analysis using MTT assay in ONH astrocytes. Note that PKA inhibition significantly promoted cell viability in ONH astrocytes treated with combined H_2_O_2_ and dbcAMP compared with ONH astrocytes treated with combined H_2_O_2_ and dbcAMP. Western blot analyses for the protein expression of pAkt S473 and pBax S184 in ONH astrocytes. Note that PKA inhibition by H89 significantly promoted protein expression of pAkt S473 and pBax S184 in ONH astrocytes treated with combined H_2_O_2_ and dbcAMP compared with ONH astrocytes treated with combined H_2_O_2_ and dbcAMP. For each determination, the protein expression in controls was normalized to a value of 1.0. Data are shown as the mean ± S.D. (*n* = 3). ∗ and ∗∗ denote *P* < 0.05 and *P* < 0.01, respectively.

**Figure 8:**
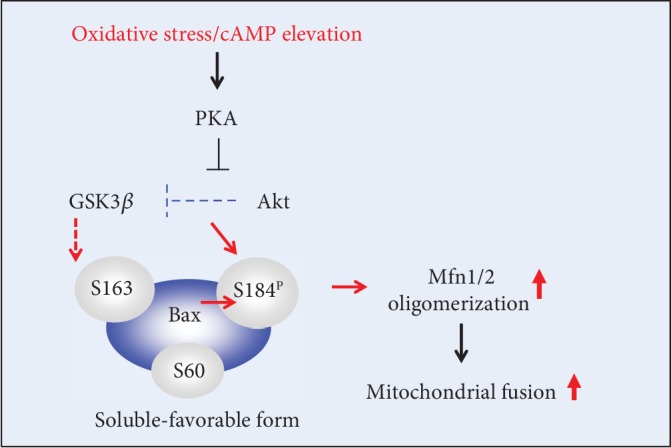
A hypothetical model for the notion that inhibition of the cAMP/PKA pathway promotes the survival of ONH astrocytes against oxidative stress combined with cAMP elevation by Akt/Bax phosphorylation-mediated Mfn1 and Mfn2 oligomerization.

We determined the effect of PKA inhibition on oligomerization of Mfn1 and Mfn2 in ONH astrocytes against oxidative stress combined with cAMP elevation. Our results demonstrated that PKA inhibition significantly increased expression levels of oligomerization of Mfn1 and Mfn2 in ONH astrocytes against oxidative stress combined with cAMP elevation (Figure 7(a)). Since PKA inhibition protects ONH astrocytes by increasing Akt phosphorylation at Ser437 against oxidative stress [7] and Akt regulates Bax phosphorylation at Ser184, inhibiting Bax effects on the mitochondria [40], we further determined the effect of PKA inhibition on Akt and Bax phosphorylation in ONH astrocytes against oxidative stress combined with cAMP elevation. We observed that PKA inhibition promoted cell viability/mitochondrial activity in ONH astrocytes against oxidative stress combined with cAMP elevation (Figure 7(b)). Our results demonstrated that PKA inhibition significantly promoted the phosphorylation of Akt at Ser437 and Bax at Ser184 in ONH astrocytes against oxidative stress combined with cAMP elevation (Figure 7(b)). Collectively, our findings suggest that inhibition of the cAMP/PKA pathway promotes the survival of ONH astrocytes against oxidative stress combined with cAMP elevation by Akt/Bax phosphorylation-mediated Mfn1 and Mfn2 oligomerization (Figure 8).

## 4. Discussion and Conclusion

We demonstrated that glaucomatous ONH astrocytes showed unbalanced mitochondrial dynamics by increasing excessive mitochondrial fission and loss, and this was accompanied by the compromised blood vessels and degenerative axons. We also observed that elevated intracellular cAMP exacerbated abnormality of mitochondrial dynamics of ONH astrocytes exposed to oxidative stress by impairing mitochondrial fusion activity. Notably, inhibition of the PKA pathway promoted the survival of ONH astrocytes against oxidative stress combined with elevated cAMP by increasing Akt/Bax phosphorylation and Mfn1/2 oligomerization.

The role of astrocytes is considered to be critical in neuroprotection or neurodestruction during glaucomatous ONH degeneration [3, 5–7, 10, 37, 41, 42]. While compromised ONH astrocytes have been reported in human patients with glaucoma and experimental animal models of glaucoma [3, 5–7, 10, 37], the pathophysiological mechanisms underlying astrocyte dysfunction in glaucomatous ONH degeneration are not fully understood. A previous study has proposed that impairment of the blood supply in the ONH by glaucomatous insult leads to the degeneration of fortified astrocytes (FASTs) that subsequently induces the restriction of FAST processes, and that this leads to axon loss by energy deprivation [5]. We have demonstrated for the first time that severe glaucoma damage triggered not only a significant loss of astrocytic processes but also degeneration of astrocytes in the glial lamina of glaucomatous DBA/2J mice [6, 7]. In the present study, our results further demonstrated the corresponding evidence of a significant loss of astrocytic processes in glaucomatous glial lamina of DBA/2J mice, accompanied by basement membrane thickening and collagen deposition in the blood vessels and axonal degeneration. Hence, we propose that elevated IOP initiates astrocyte alterations that lead to delayed ONH astrocyte dysfunction by sustained glaucomatous stress and that this dysfunctional activity of ONH astrocytes exacerbates susceptibility of axon loss by diminishing their functional support to RGC axons.

Oxidative stress links to not only mitochondrial dysfunction but also glial dysfunction during glaucoma progression [6, 10, 27, 31–33, 43–46]. Accumulating evidence indicates that vascular abnormalities induced by elevated IOP and/or hypoxia result in oxidative stress, and that leads to mitochondrial dysfunction and subsequent energy failure during glaucomatous ONH degeneration. In the ONH, human astrocytes and lamina cribrosa cells *in vitro* from patients with glaucoma showed evidence of oxidative stress, bioenergetic dysfunction, or mitochondrial dysfunction by compromising mitochondrial dynamics [6, 27]. Since glutamate excitotoxicity is a well-known source of oxidative stress, our previous study has also demonstrated that glaucomatous human ONH astrocytes from patients with glaucoma upregulated expression levels of N-methyl-D-aspartate receptors and GLAST protein, as well as promoted mitochondrial fission [6]; this suggests that oxidative stress is critical in the pathogenesis of glaucomatous optic neuropathy. Further, we have demonstrated that inhibition of oxidative stress increased mitochondrial mass and improved bioenergetic function in ONH astrocytes, while oxidative stress impaired ONH astrocytes by compromising the regulation of OXPHOS Cxs, as well as ATP production and ROS generation [9]. Nevertheless, the precise mechanism of the specific impairment in mitochondrial dynamics by which oxidative stress results in ONH astrocyte dysfunction remains elusive. In the present study, we revealed that a transient induction of oxidative stress- (relatively lower concentration of H_2_O_2_) mediated mitochondrial bioenergetic dysfunction is associated with the deficit of Drp1 protein expression, accompanied by compromised cell viability related to mitochondrial activity, but it did not show any relevance with the change of mitochondrial fusion proteins (OPA1 as well as Mfn1 and Mfn2) in ONH astrocytes. While the increase of Drp1 expression impairs neuronal cells in neurodegenerative diseases including Huntington's disease, Parkinson's disease, and glaucoma [32, 47, 48], loss of Drp1 compromises bioenergetic function in axonal mitochondria, and this leads to significant defects in maintaining the normal ATP level and synaptic vesicle cycling [49]. These collective results strongly suggest that oxidative stress-induced Drp1 defects may impair mitochondrial dynamics and subsequently trigger mitochondrial bioenergetic dysfunction in ONH astrocytes.

While the cAMP/PKA pathway has critical roles in a variety of cellular functions including cell survival and death [18, 19, 50, 51], there is surprisingly little known to date about the cAMP/PKA pathway-mediated pathophysiological signaling mechanism in injured ONH astrocytes during glaucomatous neurodegeneration. Emerging evidence from our group unveiled a novel degenerative signaling mechanism that activation of the intracellular cAMP/PKA signaling pathway leads to the impairment of ONH astrocytes by inhibiting Akt phosphorylation and activating Bim/Bax activation and that this leads to caspase-3 activation-mediated cell death [7]. Notably, this finding was strongly supported by increasing expression of cAMP and active Bax and caspase-3 proteins in the ONH astrocytes of glaucomatous DBA/2J mice [7]. Therefore, these findings suggest that activation of intracellular cAMP signaling cascade is an important pathophysiological mechanism in ONH astrocyte dysfunction and its significance should be considered in the future studies of glaucomatous ONH degeneration.

While accumulating evidence links cAMP signaling to mitochondrial dynamics in mammalian cells, this connection in ONH astrocytes remains unexplored. In contrast to oxidative stress-induced Drp1 loss in ONH astrocytes, we here demonstrated that the elevated intracellular cAMP level increased mitochondrial fission Drp1 protein expression, but not mitochondrial fusion proteins such as OPA1as well as Mfn1 and Mfn2. These results raise the possibility that Drp1 critically contributes to the alteration of mitochondrial dynamics of ONH astrocyte dysfunction in response to oxidative stress and/or intracellular cAMP elevation. Based on our recent finding that elevated cAMP exacerbates vulnerability to oxidative stress in ONH astrocytes [7], we further addressed the effect of elevated cAMP on mitochondrial dynamics in ONH astrocytes exposed to oxidative stress. Our results demonstrated that elevated cAMP induced a significant reduction of total OPA1 as well as Mfn 1 and Mfn2 protein expression in ONH astrocytes exposed to oxidative stress, while total Drp1 protein expression was kept at a control level. Indeed, this surprising alteration of mitochondrial dynamics was accompanied by a worse reduction of cell viability related to mitochondrial activity in ONH astrocytes. Thus, these results suggest that elevated intracellular cAMP level accelerates mitochondrial dysfunction of ONH astrocytes exposed to oxidative stress through the impaired activity of mitochondrial fusion, leading to ONH astrocyte dysfunction and degeneration.

We have previously proposed that impairment of mitochondrial dynamics is linked to ONH astrocyte dysfunction in glaucomatous neurodegeneration [6, 9]. Indeed, the increased excessive fission-mediated mitochondrial dysfunction was present in glaucomatous human and mouse ONH astrocytes, as well as in oxidative stress-induced rat ONH astrocytes *in vitro* [6, 9]. Nevertheless, there is no evidence for impaired mitochondrial fusion activity in glaucomatous ONH astrocytes. Mfn1 and Mfn2 are GTPase dynamin-like proteins of the outer mitochondrial membrane, which are essential for fusion activity in the mitochondria of human cells [52–54]. While mutations of Mfn2 compromise motor and sensory neurons in Charcot-Marie-Tooth disease [55], overexpression of Mfn2 protects neuronal cells in the brain against hypoxia-induced apoptosis or ischemia/reperfusion [56, 57]. In addition, activated Mfn2 protects the mitochondria by inhibiting Bax activation, cytochrome c release, and permeability transition [58]. Recent evidence has reported the decrease of Mfn1 and Mfn2 protein expression in the ON of aged glaucomatous DBA/2J mice [59], raising the possibility that altered expression levels of Mfn1 and Mfn2 proteins may contribute to glaucomatous ONH degeneration. In the present study, we demonstrated for the first time that elevated cAMP induced loss of Mfn1 and Mfn2 in ONH astrocytes exposed to oxidative stress. More importantly, PKA inhibition restored mitochondrial morphology and promoted ONH astrocyte survival by increasing the oligomerization of both Mfn1 and Mfn2 against oxidative stress combined with cAMP elevation. Thus, our findings suggest that increasing the activity of Mfn1 and Mfn2 oligomerization may have therapeutic potential to protect ONH astrocytes against glaucomatous insults.

Reduction of cAMP protects rat cortical astrocytes by activating Akt pathway *in vitro* [60–62]. Indeed, emerging evidence from our group revealed that the activation of the cAMP/PKA signaling pathway was critical to the vulnerability of oxidative stress-induced ONH astrocytes and that inhibition of this activation of the cAMP/PKA pathway promoted the survival of ONH astrocytes via increasing Akt phosphorylation at Ser437 against oxidative stress [7]. Notably, we here demonstrated that PKA inhibition by H89 induced a significant increase of not only Akt phosphorylation at Ser437 but also Bax phosphorylation at Ser184 in ONH astrocytes against oxidative stress combined with cAMP elevation. Akt inhibits a conformational change in the Bax protein and its translocation to mitochondria, leading to prevent mitochondrial dysfunction and cell death [63]. Further, Akt regulates Bax phosphorylation at Ser184 which, in turn, inhibits Bax effects on the mitochondria [40]. Since we have proposed a link of the pathological pathway between cAMP/PKA activation and Akt/Bim/Bax-mediated intrinsic cell death in ONH astrocyte dysfunction [7], our results strongly suggest the notion that Akt/Bax phosphorylation by inhibiting the cAMP/PKA pathway would be an important defense mechanism in glaucomatous ONH astrocyte dysfunction.

Although H89 is a well-known PKA inhibitor, we propose that the use of H89 should be carefully interpreted due to its nonspecific inhibitory effect on other kinases including ribosomal S6 kinase 1 (S6K1). Indeed, H89 also inhibits S6K1 (80 nM) at lower IC50 than PKA (135 nM) in a kinase assay *in vitro* [64]. Hence, H89 could be used in conjunction with other PKA inhibitors, such as Rp-cAMPS or PKA analogs, to investigate the effect of PKA [65, 66]. We have previously reported that PKA inhibition by H89 restored the level of Akt phosphorylation by forskolin, an adenylyl cyclase activator. Moreover, PKA inhibition by PKI, a potent competitive synthetic peptide PKA inhibitor, increases Akt phosphorylation in both normal and oxidative stress conditions [7], demonstrating that PKA mediates Akt phosphorylation in ONH astrocytes. However, we cannot exclude the possibility that a direct inhibition of S6K1 by H89 may affect the level of Akt phosphorylation in ONH astrocytes because S6K1 inhibition by PF-4708671, a selective S6K1 inhibitor, also increases the level of Akt phosphorylation in insulin treated-hepatocytes, but not in the normal condition [67]. Therefore, it would be useful to determine whether H89 regulates Akt phosphorylation via solely PKA or in combination with other kinases such as S6K1 in future studies.

We have previously raised a possibility that a transient induction of oxidative stress with a relatively lower concentration of H_2_O_2_ (50 *μ*M) triggers an endogenous mechanism by reducing the intracellular level of cAMP in ONH astrocytes. In similar way, the result of the lower level of Bax protein expression by H_2_O_2_ (50 *μ*M) treatment (Figure 5(b)) also raises a possibility that an endogenous compensatory mechanism induced by a lower concentration of H_2_O_2_ may trigger the reduction of Bax protein expression in ONH astrocytes. While Bax does not alter the activity of Mfn1-Mfn2 *trans* heterotypic complexes, soluble Bax positively regulates mitochondrial fusion activity by Mfn2 homotypic complexes on mitochondria. It has been suggested that cytoplasmic Bax is endogenously phosphorylated most likely at Ser184, regulating heterodimerization of Bax with antiapoptotic Bcl-2 family members [40]. Since Bax is phosphorylated only in the cytoplasm, the soluble [40], nonoligomerized form of Bax is the primary cytosolic regulator of mitochondrial fusion [68]. Thus, our findings also suggest that the cAMP/PKA-mediated Akt/Bax pathway may control mitochondrial dynamics via regulating oligomerization activity of Mfn1 and Mfn2 in ONH astrocytes against oxidative stress.

In conclusion, we provide evidence that the activation of the cAMP/PKA pathway has a critical role in the impairment of mitochondrial dynamics and bioenergetics of ONH astrocytes. Moreover, elevated cAMP exacerbates mitochondrial dysfunction to oxidative stress of ONH astrocytes. Inhibition of the intracellular cAMP/PKA pathway can protect ONH astrocytes by increasing Akt/Bax phosphorylation and Mfn1 and Mfn2 oligomerization. Therefore, we suggest that modulation of the cAMP/PKA pathway may have therapeutic potential to protect ONH astrocytes by preserving the mitochondrial network and function in glaucomatous ONH degeneration.

## Figures and Tables

**Table 1:** Primer sequences.

	Forward primer	Reverse primer
Drp1	cgaaaactgtctgcccgaga	gctgccctactaattcactc
Mfn1	gtgaagttcacaagtgcaaa	gctcgggtggagaaactgct
Mfn2	gctgacctggaccaccagag	tggctttgctctgaagtgaa
OPA1	gagtcgaagtcgatccaagc	cgcctaacttcggtgtttgt
GAPDH	agaacatcatccctgcatcc	gtcctcagtgtagcccagga

## Data Availability

All the data used to support the findings of this study are included within the article.
