# Corrigendum to “Inhibition of cAMP/PKA Pathway Protects Optic Nerve Head Astrocytes against Oxidative Stress by Akt/Bax Phosphorylation-Mediated Mfn1/2 Oligomerization”

**DOI:** 10.1155/2020/9410289

**Published:** 2020-09-30

**Authors:** Won-Kyu Ju, Myoung Sup Shim, Keun-Young Kim, Tae Lim Park, Sangphil Ahn, Genea Edwards, Robert N. Weinreb

**Affiliations:** ^1^Viterbi Family Department of Ophthalmology, Hamilton Glaucoma Center and Shiley Eye Institute, University of California San Diego, La Jolla, California, USA; ^2^Department of Ophthalmology, Duke University, Durham, North Carolina, USA; ^3^National Center for Microscopy and Imaging Research and Department of Neuroscience, University of California San Diego, La Jolla, California, USA

In the article titled “Inhibition of cAMP/PKA Pathway Protects Optic Nerve Head Astrocytes against Oxidative Stress by Akt/Bax Phosphorylation-Mediated Mfn1/2 Oligomerization” [1], there was an error in Materials and Methods and Results where “Ser437” was used instead of “Ser473.” Materials and Methods and Results are shown below with this error corrected.

## 2. Materials and Methods

### 2.9. Western Blot

Cells were harvested and lysed for 30 min on ice with a modified RIPA lysis buffer (150 mM NaCl, 1 mM EDTA, 1% NP-40, 0.1% SDS, 1 mM DTT, 0.5% sodium deoxycholate, and 50 mM Tris-Cl, pH 7.6), containing complete protease inhibitors. The lysates were centrifuged at 15000 g for 15 min, and the protein amounts in the supernatants were measured by Bradford methods. Proteins (5-10 *μ*g) were separated by SDS/PAGE and electrotransferred to the PVDF membrane. The membrane was blocked with 5% nonfat dry milk and PBS/0.1% Tween-20 (PBS-T) for 1 h, incubated with primary antibodies overnight at 4°C. Primary antibodies are rabbit polyclonal anti-Akt antibody (1 : 5000; Cell Signaling, Danvers, MA, USA), mouse monoclonal anti-phospho-Akt (Ser473) antibody (S473, 1 : 2000; Cell Signaling), mouse monoclonal anti-Bax antibody (N-20; 1 : 2000; Santa Cruz Biotechnology, Santa Cruz, CA, USA), rabbit polyclonal anti-phospho-Bax (Ser184) antibody (1 : 2000; Cell Signaling), mouse monoclonal anti-Drp1 antibody (1 : 5000; BD Biosciences), mouse monoclonal anti-OPA1 antibody (1 : 5000; BD Biosciences), mouse monoclonal anti-Mfn1 and anti-Mfn2 antibody (1 : 3000; Abcam, Cambridge, UK), and mouse monoclonal anti-actin antibody (1 : 100,000; Millipore, Burlington, MA, USA). After several washes in PBS-T, the membranes were incubated with peroxidase-conjugated goat anti-mouse or rabbit IgG (1 : 5000; Bio-Rad, Hercules, CA, USA) and developed using an enhanced chemiluminescence substrate system. The images were captured and quantified by using an ImageQuant™ LAS 4000 system (GE Healthcare Bio Science), and the band densities were normalized to the band densities for actin.

## 3. Results

### 3.6. PKA Inhibition Promotes Oligomerization of Mfn1 and Mfn2 and Phosphorylation of Akt and Bax in ONH Astrocytes against Oxidative Stress and cAMP Elevation

We determined the effect of PKA inhibition on oligomerization of Mfn1 and Mfn2 in ONH astrocytes against oxidative stress combined with cAMP elevation. Our results demonstrated that PKA inhibition significantly increased expression levels of oligomerization of Mfn1 and Mfn2 in ONH astrocytes against oxidative stress combined with cAMP elevation (Figure 7(a)). Since PKA inhibition protects ONH astrocytes by increasing Akt phosphorylation at Ser473 against oxidative stress [7] and Akt regulates Bax phosphorylation at Ser184, inhibiting Bax effects on the mitochondria [40], we further determined the effect of PKA inhibition on Akt and Bax phosphorylation in ONH astrocytes against oxidative stress combined with cAMP elevation. We observed that PKA inhibition promoted cell viability/mitochondrial activity in ONH astrocytes against oxidative stress combined with cAMP elevation (Figure 7(b)). Our results demonstrated that PKA inhibition significantly promoted the phosphorylation of Akt at Ser473 and Bax at Ser184 in ONH astrocytes against oxidative stress combined with cAMP elevation (Figure 7(b)). Collectively, our findings suggest that inhibition of the cAMP/PKA pathway promotes the survival of ONH astrocytes against oxidative stress combined with cAMP elevation by Akt/Bax phosphorylation-mediated Mfn1 and Mfn2 oligomerization (Figure 8).
